# Supplementary figures and images for: Female reproductive tract has low concentration of SARS-CoV2 receptors
Source: PLoS One. 2020 Dec 14;15(12):e0243959. doi: 10.1371/journal.pone.0243959 (PMC7735593; doi:10.1371/journal.pone.0243959)

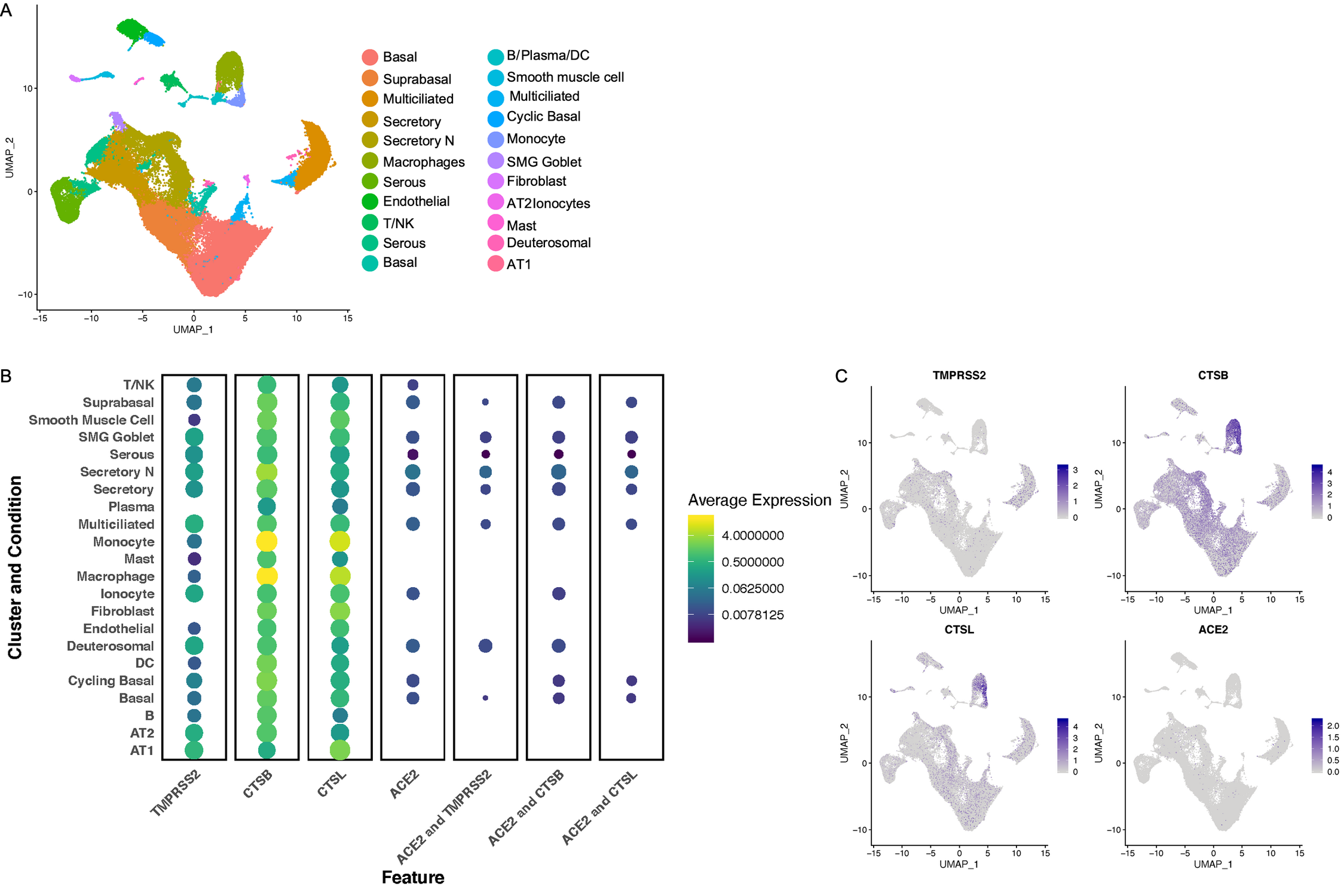

Supplement: S1 Fig — A) UMAP showing the cell annotation of the human lung. B) Dot plots showing the expression of the genes in each cell type along with the co-expression of the ACE2/TMPRSS2, ACE2/CTSB and ACE2/ CTSL in the lung (with Benjamini–Hochberg-adjusted p values). The dot size represents the proportion of the cells within the respective cell type expressing the gene and the color indicates the average gene expression. C) Feature plots showing the expression of SARS-CoV2 receptor, ACE2, and proteases TMPRSS2, CTSB and CTSL in the lung. Grey: No RNA expression purple: RNA positive. (TIF) [file pone.0243959.s001.tif]

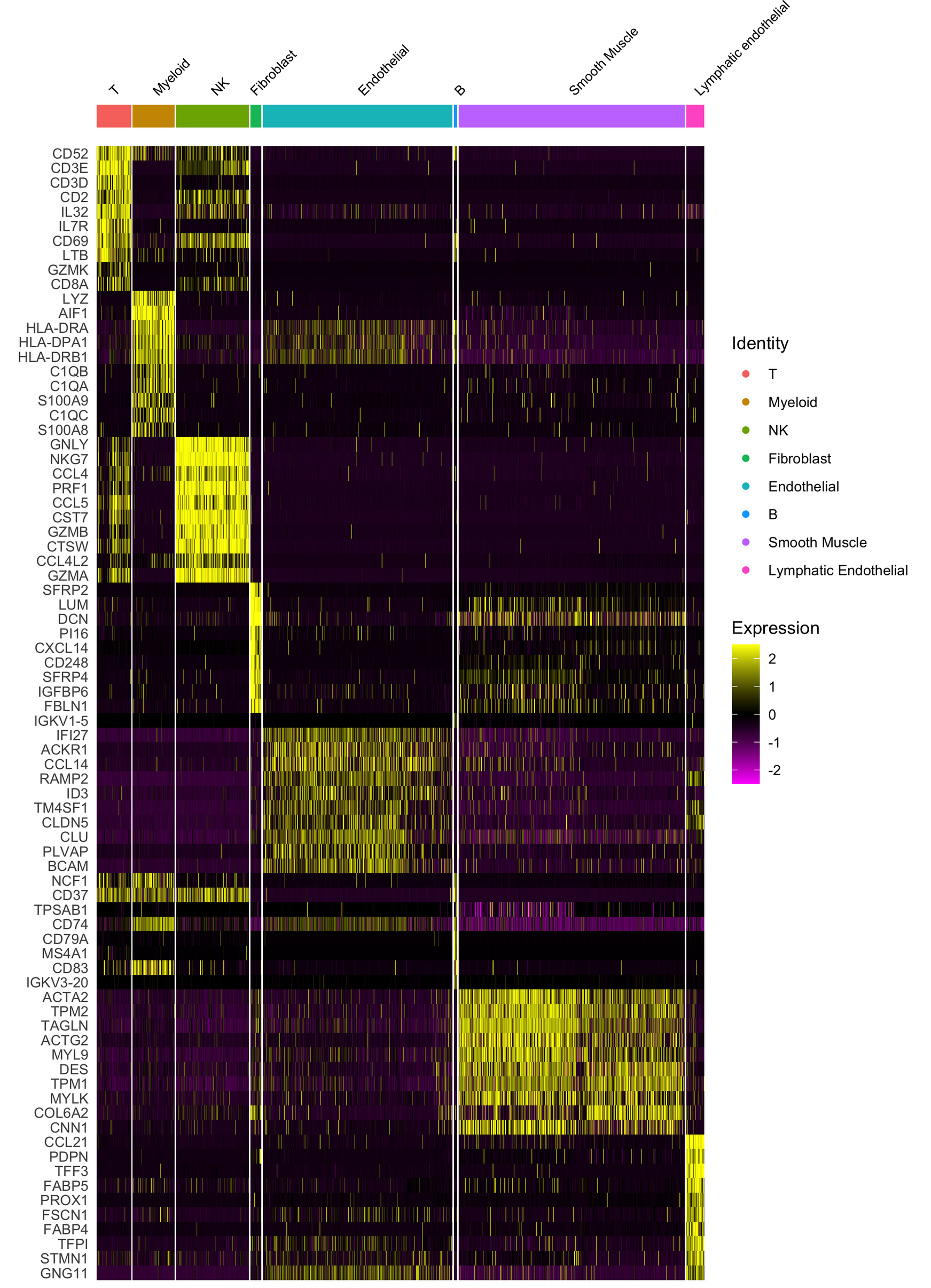

Supplement: S2 Fig — Colors represent the expression level as shown in the scale bar. (TIF) [file pone.0243959.s002.tif]
